# Rediscovering tandem repeat variation in schizophrenia: challenges and opportunities

**DOI:** 10.1038/s41398-023-02689-8

**Published:** 2023-12-20

**Authors:** Rebecca Birnbaum

**Affiliations:** 1 Department of Psychiatry, Icahn School of Medicine at Mount Sinai, New York, NY USA; 2 Department of Genetics and Genomics Sciences, Icahn School of Medicine at Mount Sinai, New York, NY USA

**Keywords:** Genomics, Molecular neuroscience, Clinical genetics

## Abstract

Tandem repeats (TRs) are prevalent throughout the genome, constituting at least 3% of the genome, and often highly polymorphic. The high mutation rate of TRs, which can be orders of magnitude higher than single-nucleotide polymorphisms and indels, indicates that they are likely to make significant contributions to phenotypic variation, yet their contribution to schizophrenia has been largely ignored by recent genome-wide association studies (GWAS). Tandem repeat expansions are already known causative factors for over 50 disorders, while common tandem repeat variation is increasingly being identified as significantly associated with complex disease and gene regulation. The current review summarizes key background concepts of tandem repeat variation as pertains to disease risk, elucidating their potential for schizophrenia association. An overview of next-generation sequencing-based methods that may be applied for TR genome-wide identification is provided, and some key methodological challenges in TR analyses are delineated.

## Introduction

Schizophrenia is a severe psychiatric disorder of public health import affecting approximately 1% of the global population, characterized by a triad of symptoms, “positive” (e.g., delusions and hallucination), “negative” (e.g., social withdrawal and avolition) and cognitive (e.g., executive dysfunction) [1]. Schizophrenia is caused by a combination of both genetic and environmental factors, with past family and twin studies estimating heritability up to 80% [2, 3]. Arguably, the landscape of genomic discovery in schizophrenia has shifted dramatically within the past decade, from metaphoric “famine” to “feast”, driven by scale, the ability to amass large schizophrenia cohorts and to sequence or genotype at high and relatively unprecedented throughput. Consequently, recent large-scale schizophrenia genetic association studies, enabled by collaborative multi-site consortia, have resulted in the identification of credible risk loci throughout the genome, overcoming past decades of relatively unsuccessful or inconsistent attempts [4–6].

The evolution of different classes of genomic markers has driven disease mapping efforts for complex disorders, including schizophrenia, since the 1980s (Table 1) [7]. Initial disease mapping attempts in the 1980s and 1990s utilized restriction fragment length polymorphisms (RFLPs) and short tandem repeat variation (i.e., microsatellite markers) distributed throughout the genome to discover genomic regions underlying risk [8, 9]. The density of these microsatellite markers was sparse, however, and assays to detect their polymorphisms were limiting and low throughput [10]. Initial linkage analyses and candidate gene studies for schizophrenia were relatively small scale, notable especially for inconsistency and lack of replication [11, 12]. Single-nucleotide polymorphic (SNP) markers emerged in the early 2000s as alternative genomic markers for more cost-effective and higher throughput association studies, supplanting previously used genomic markers and yielding significant associations for many disorders, including for neuropsychiatry [13]. For example, in the largest schizophrenia common variant genome-wide association study (GWAS) meta-analysis published to date, assaying single-nucleotide polymorphisms (SNPs) in 76,755 individuals with schizophrenia and 243,649 control individuals, the Psychiatric Genomics Consortium identified 287 statistically significant, independent loci, while a polygenic combination of SNPs across the genome explained up to 8% of schizophrenia liability [4]. In addition to SNPs, the investigation of other classes of genomic variants has resulted in the identification of rare variant risk factors, of greater effect size than common SNPs, found in a small minority of affected individuals, likely 1–3% of schizophrenia cases. For example, eight highly penetrant large structural variants in the genome, copy number variations, were reported as significantly associated with schizophrenia (odds ratios of 4–68) in an analysis of 21,094 schizophrenia cases and 20,227 controls, while rare single-nucleotide coding variants within ten genes were found to confer substantial risk to schizophrenia (odds ratios of 3–50) in exome sequencing analyses of 24,248 schizophrenia cases and 97,322 controls [5, 6, 14, 15].

**Table 1:** Evolution of genomic markers for disease mapping/discovery of risk loci for complex disorders, including schizophrenia.

	1980s/1990s	2000s	2010s	2020s??
Genomic marker	-RFLPs (restriction fragment length polymorphisms) -“Microsatellites” (short tandem repeats)	SNPs (single-nucleotide polymorphism)	SNVs (single-nucleotide/protein-coding variants)	?Re-discovering -“Microsatellites” (short tandem repeats) and -“Macrosatellites” (variable number tandem repeats) -Other structural variants
Population genomics reference	Genetic linkage maps ~400 markers (1987) ~5000 microsatellite markers (1996)	Catalogued SNPs/linkage disequilibrium 1 million SNPs (2005, HapMap) 3 million SNPs (2007, HapMap)	SNVs catalogued in reference genome ~10,000 SNVs (2008)	Tandem repeats catalogued in reference genome ~1 million tandem repeats (tandem repeat finder, 2022)
Study design	Linkage analyses/candidate gene approach of genotyped cohorts	Genome-wide association studies of genotyped cohorts	Genome-wide profiling of whole-exome-sequenced cohorts	Genome-wide profiling of whole-genome-sequenced cohorts
Schizophrenia (SCZ) application/example	Numerous reports, inconsistent findings and overall lack of replication	Psychiatric Genomics Consortium, 2014: 36,989 SCZ cases; 113,075 CONT 108 significant independent loci 2022: 76,755 SCZ cases, 243,649 CONT 287 significant independent loci	Schizophrenia Exome Sequencing Consortium 2022: 24,248 SCZ cases, 97,322 CONT 10 significant genes with coding variants	

An overview of the evolution of genomic markers used in complex disease studies since the 1980s, indicating the class of marker by decade, the initial marker density in the reference genome, relevant study designs incorporating the marker, and schizophrenia application.

Numerous post-GWAS studies are ongoing to begin to translate and advance the clinical saliency of emerging schizophrenia genomics findings. Functional genomic studies across tissue and cell-type aim to elucidate specific causal variants or molecular species within reported schizophrenia risk loci, fine-mapping and gene-prioritization strategies are being deployed, and polygenic risk scores are being investigated for clinical stratification [4, 16–22]. A fundamental remaining conundrum however is the “missing heritability” of schizophrenia. Genetic association studies to date still explain only a small part of the putative heritable risk for schizophrenia (and other complex diseases), while most predicted heritability remains “unexplained”, suggesting that other genetic risk factors, genetic interactions or other classes of genomic variants may still contribute to schizophrenia risk.

Tandem repeat variation is one such prominent class of genomic variant, currently under-assayed compared to SNPs that, though incorporated in earlier genomic studies, has been largely excluded from more recent large-scale schizophrenia genomic association studies. Yet, the high mutation rate of tandem repeats, often orders of magnitude higher than SNPs and indels, indicates that they are very likely to make significant contributions to phenotypic variation. The high-throughput profiling of tandem repeat variation has become increasingly feasible with the advent of a suite of recently developed, next-generation sequencing-based tools and facilitated by the increased density of the reference genome, containing more than one million cataloged tandem repeats. Therefore, the current investigation of tandem repeat variation may indeed be highly informative for schizophrenia (and other neuropsychiatric disorders).

## Known clinical/phenotypic effects of tandem repeat variation

Tandem repeats (TRs) are stretches of DNA comprised of two or more contiguous repeats of a sequence of nucleotides arranged in a head-to-tail pattern. TRs are prevalent throughout the genome, with more than 1 million TR loci currently annotated, constituting at least 3% of the genome, and located ubiquitously in untranslated regions, but also in coding regions and regulatory regions, including promoters and enhancers [23, 24]. TRs range in motif size and are categorized as short tandem repeats (STRs) or “microsatellites”, with motif lengths of 1–6 bp, for example, mono-nucleotide repeats (e.g., GGGGGG) or trinucleotide poly(CAG) repeats. TRs with longer motifs (≥7 bp repeated in tandem) are termed variable number tandem repeats (VNTRs) or “minisatellites”, in some cases even containing entire exons or genes within each repeated unit [23–25]. TRs may exhibit variation that is rare or common in frequency, and in contrast to (biallelic) SNPs, TR variation may be multi-allelic. Due to their repetitive nature, which can induce frequent errors in recombination and replication, TRs are among the most polymorphic markers of the genome, i.e., they often show high mutation frequencies, with many multi-allelic TRs showing high levels of length polymorphism, even within a single family or within different cells in an individual [25, 26].

### Rare tandem repeat variation and disease risk

Rare variation arising from meiotic instability may cause extreme changes in length; for example, some TR loci occasionally expand to contain hundreds or even thousands of additional copies compared to that found in the general population, in coding or non-coding regions of the genome [27–29]. These rare tandem repeat expansions (TREs), most commonly observed at STR loci, were first discovered over 30 years ago, and are now known to underlie more than 50 different human diseases, including neurodevelopmental disorders, such as Fragile X syndrome, and late-onset neurodegenerative disorders, such as Huntington disease and amyotrophic lateral sclerosis (ALS) [27, 28, 30]. Thus, TREs are an established, heritable mutational mechanism that contribute to a variety of human disease, most frequently and interestingly, observed to date in disorders affecting the central nervous system. Disease-causing TR expansions can be located in gene promoters, 5’ and 3’ untranslated regions, introns, or protein-coding exons, often in coding, triplet repeat poly(CAG) regions. The mechanism of pathogenicity varies by TRE, including loss-of-function through transcription silencing, RNA-mediated gain-of-function through RNA-binding protein sequestration, and repeat-associated translation of toxic peptides [31].

### Common tandem repeat variation and disease risk

In addition to rare TR variation, common TR variation is increasingly being implicated in complex disease associations. As an example of an already known association, for the highly heritable trait of lipoprotein (a) concentration, the elevation of which is a major risk factor for cardiovascular disease, half of the population variance may be explained by variation in VNTR copy number in the second kringle-IV (KIV) domain of *LPA*; longer alleles with more copies of the kringle repeat are associated with lower lipoprotein (a) levels [32]. In a recent UK Biobank analysis of 786 phenotypes among 415,280 participants, a subset of 118 coding VNTR polymorphisms were strongly associated with multiple phenotypes, corroborating the association of lipoprotein concentration with LPA copy number, as well as identifying other, novel associations of VNTR variation with height, hair morphology, kidney function, and other cardiac phenotypes [33]. Furthermore, within some GWAS-positive loci, VNTRs have been found to be more strongly associated with complex traits than the previously reported lead index SNPs. An alternative large-scale phenome-wide association analysis of 283 traits in ~35,000 whole-genome sequenced individuals from the NHLBI *TOPMed*, profiled ~55,000 common VNTR polymorphic variants, genome-wide in coding and non-coding regions, identifying 21 significant complex trait-VNTR association findings, including confirmation of the previously reported association of *ACAN* VNTR copy number with height [34]. Notably, these initial large-scale VNTR-phenotype association analyses excluded schizophrenia as a complex trait, however, instead focusing on other more prevalently recorded traits, such as markers of cardiometabolic disorders and height.

## A role for tandem repeat variation in schizophrenia risk?

Limited investigations of tandem repeat variation and schizophrenia to date as summarized below suggest a role of TR variation in schizophrenia, both rare and common in frequency.

### Rare tandem repeat variation and schizophrenia risk

In a series of one-off case reports, in patients with features of psychosis or schizophrenia, an empiric strategy of targeted sequencing of select genes known to be associated with neurological disorders identified rare, repeat expansions (for example, in *HTT*, *ATXN8OS* and *C9orf72*, known to be associated with Huntington’s disease (HD), hereditary ataxias, and amyotrophic lateral sclerosis, respectively) [35–39]. A broader, genome-wide analysis of TREs in a modest-sized cohort of 257 individuals with schizophrenia compared to 2729 controls, identified TREs in individuals with schizophrenia involving 193 genes, including TREs in intronic and exonic regions, and several TREs proximal to schizophrenia GWAS loci (<10 kb), though the penetrance of the reported TREs could not be determined [40]. In the largest study to date, analyzing TREs in 1154 schizophrenia cases compared to 934 matched controls, up to 603 potential TREs were identified, found to be enriched in brain eQTLS and in genes differentially expressed in brain-specific schizophrenia analyses, though overall, the authors acknowledge the study was underpowered to detect TREs significantly associated with schizophrenia, genome-wide [41]. Although these studies were unable to determine whether any of these potential TREs definitively contribute to schizophrenia risk, the identification of these TREs and their apparent enrichment at loci previously associated with schizophrenia and brain function suggest potential disease relevance.

Since anticipation may occur in tandem repeat expansion disorders, notably, a few analyses of anticipation in schizophrenia were published during the linkage era (mostly in the 1990s), with discrepant findings, testing for an inheritance pattern in which disease severity increases, or age-of-onset decreases, in successive generations [42–45]. For example, in a study of 137 intergenerational pairs of schizophrenia, a median of 15 years earlier in age of onset of illness was reported for the younger generation, using first admission to psychiatric hospital as a proxy for age-of-onset [42]. An alternative analysis of registries from the UK and the United States found a median age of onset difference of 8 years earlier in the younger generation in 101 intergenerational pairs affected by schizophrenia spectrum disorders [44]. Overall, previous reports of anticipation in schizophrenia were limited by pedigree size and, furthermore, critiqued for ascertainment bias, including over-recruitment of parents at late age (as fertility could be reduced in patients with early age-of-onset), as well as a bias in preferential ascertainment of offspring with an earlier age-of-onset [43, 44, 46]. Going forward, as enabled by large-scale TRE profiling, if rare tandem repeat expansions at novel loci are identified as “pathogenic” for some fraction of schizophrenia cases, then the potential correlation of TREs with severity of symptoms or age-of-onset may be meaningful to query within familial registries or quad or trios study designs.

### Common tandem repeat variation and schizophrenia risk

**Fig. 1:**
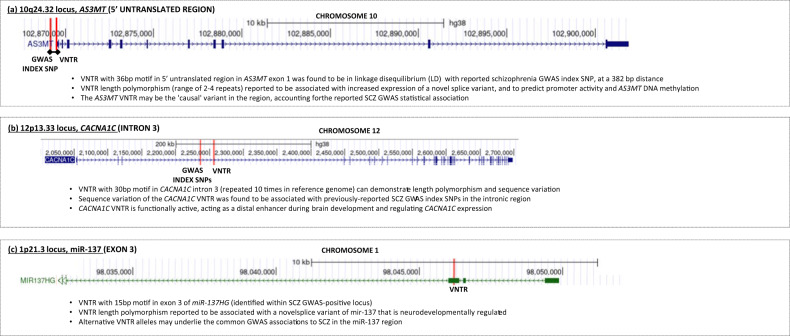
Tandem repeat elements influencing schizophrenia risk, within schizophrenia GWAS-positive loci. For each of the three schizophrenia GWAS-positive loci, the mechanism and genomic location of a VNTR reported to influence schizophrenia risk is indicated.

In addition to rare tandem repeat expansions in schizophrenia, several recent studies have identified common tandem repeat variation, VNTRs, that influence schizophrenia risk within GWAS-positive loci (Fig. 1): (i) within a highly ranked schizophrenia GWAS-positive locus, 10q24.32, a VNTR in exon 1 of *AS3MT* was found to be in linkage with the GWAS index SNP and to have a functional effect on *cis-*gene expression [47]. The study suggested that GWAS index SNP was in effect a proxy for the VNTR, which was likely the “causal” variation at this locus: (ii) within another highly ranked schizophrenia GWAS-positive locus, a 30 bp VNTR was identified in the third intron of *CACNAC1C*, a gene encoding a subunit of the L-type calcium channel, of widespread neurobiological import [48]. Different VNTR alleles were functionally associated with modification of transcriptional enhancer activity and altered schizophrenia risk. (iii) Within the GWAS-positive locus spanning the microRNA gene (*MIR137HG*), a 15-bp VNTR was identified to effect miR-137 alternative splicing and to contribute to schizophrenia risk [49]. Overall, while initial studies suggest a role for TR variation in schizophrenia, more robust, systematic and genome-wide studies are needed to elucidate and instantiate their role in schizophrenia genomic risk.

### Tandem repeat variation and other psychiatric disorders

By comparison, robust TR analyses of other complex psychiatric disorders have yet to emerge, except for initial reports characterizing TRs in some ASD cohorts. A genome-wide analysis of de novo STR mutations in 1637 quad simplex families (individuals with ASD and their unaffected family members) in the Simons Simplex Collection revealed a significant excess of STR mutations in ASD probands, though the overall burden analysis was underpowered to detect specific TR loci enriched for mutations in probands versus siblings at genome-wide significance [50]. Furthermore, mutations showed a bias towards expansions (71%) versus contractions (29%), and with phasing indicating that the expansion bias was driven by maternally derived mutations. Another genome-wide analysis queried TR expansions in genomes from autism families and population controls (8448 samples from MSSNG project, 9096 samples from Simons Simplex Collection, 2504 samples from 1000 Genomes project), reporting an increased rate of TR expansions in autism-affected children (23.3%) compared to unaffected children (20.7%), along with the identification of TREs in known risk genes, such as *DMPK* and *FXN* and within many novel loci, such as *CACNB1* [51]. Interestingly, ASD-associated rare TREs were found to be increased in exonic and splicing regions, suggesting potential regulatory roles. These initial reports of TR variation in autism-affected individuals are larger in scale than the TR analyses schizophrenia cohorts, reported to date.

## Challenges and opportunities in sequencing tandem repeat variation

Overall, tandem repeats remain poorly studied compared to other classes of variants, more difficult to assay than SNPs and typically excluded from microarray designs due to their high-copy nature [52]. As pertains to under-ascertainment in recent GWAS studies, due to their multi-allelic nature and high mutation rate, most tandem repeat variants are thought to be poorly tagged by nearby SNPs [53, 54]. The relative dearth of systematic characterization of phenotypic consequence of TR variation is therefore largely attributable to past technical difficulties in high-throughput, reliable TR genotyping (i.e., determining TR length or number of repeating units). TR genotyping has been highly error-prone due to various sequencing challenges, including extreme GC content, accurate alignment due to variation in TRs appearing as large insertions or deletions relative to the reference, and PCR-based “stutter noise” or artificial variability in the sequence [55, 56]. Furthermore, while some TR imputation panels from genotype array data have been developed, accurate TR genotyping requires access to whole-genome sequence (WGS) data, relatively scarce in past years, but becoming increasingly available for some schizophrenia cohorts [57–59]. Specialized approaches to sequence tandem repeats are often required even for WGS data, due to the difficulty of mapping and interpreting reads in non-unique and highly variable parts of the genome. A suite of publicly available next-generation sequence (NGS)-based TR-profiling methods have been developed in recent years that may be applied to profiling TRs in schizophrenia WGS cohorts, each method with comparative advantages and disadvantages, and with each tool utilizing different computational/analytical strategies, meaning that each has differing sensitivities depending on the sequence characteristics of each TR locus being analyzed (see Tables 2 and 3).

**Table 2:** Tools for genotyping STRs.

STR genotyping tool	Algorithm description	Genotype TRs that exceed the read limit?	Detects TRs not annotated in reference?	Other notes/features
HipSTR [62]	Learns a parametric model that captures each STR’s stutter noise profile. Using the genomic location of the repeat, harnesses this profile and a hidden Markov model (HMM) to realign the STR-containing reads to candidate haplotypes, mitigating the effects of PCR stutter	No	No	Reliability: multiple publications have used HipSTR as singular tool, i.e., reports of case status associations (in ASD Simons Simplex Collection), or eQTL analyses (GTEx); can phase STRs
lobSTR [63]	Signal processing approach that uses rapid entropy measurements to find informative STR reads followed by a Fast Fourier Transform to characterize the repeat sequence	No	No	High error rates noted for dinucleotide repeats
STRetch [69]	Remaps reads anchored in the vicinity of a putative TRE to a synthetic decoy genome containing large expanded repeat arrays; considers reads that map preferentially to synthetic decoy genomes as major criterion in scoring algorithm	Yes	No	Incorporates an outlier statistical method in identifying expansions
gangSTR [70]	Relies on a statistical model incorporating multiple properties of paired-end reads into a single maximum likelihood framework capable of genotyping both normal length and expanded repeats	Yes	No	Uses an exhaustive grid search over all possible allele pairs and returns the maximum likelihood diploid genotype
Expansion Hunter [71]	Sequence-graph-based realignment of reads that originate inside and around each target repeat. Genotypes the length of the repeat in each allele based on these graph alignments	Yes	No	Expansion bias, Repeats with long motifs may gain evidence for expansion
Expansion Hunter DeNovo (102) [76]	Counts number of anchored in-repeat reads (IRRs), which are read pairs in which the first read (the IRR) contains repetitive sequence and the second read (the anchor) contains non-repetitive sequence that can be uniquely mapped to the reference genome	Yes	Yes	TRE must be larger than the sequence read length (>100–150 bp) to be detected
STRling [77]	Performs k-mer counting in DNA sequencing reads, to efficiently recover reads that inform the presence and size of STR expansions	Yes	Yes	Pending replication studies
exSTRa [72]	Generates empirical cumulative distribution functions (ECDFs) of repeat-motif distributions	Yes	No	May be advantageous in WES data
Tredparse [74]	Probabilistic model for predicting STR lengths on the basis of evidence from spanning reads, partial reads, repeat-only reads, and spanning pairs	Yes	No	Does not detect expansions that exceed its detection threshold
superSTR [75]	Uses a fast, compression-based estimator of the information complexity of individual reads to select and process only reads likely to harbor repeat expansions for processing using the linear-time maximal repetition detection algorithm	Yes	Yes	Does not require alignment of raw sequence data

Several publicly available tools for genotyping STRs from whole-genome-sequence data are tabulated, along with notes on the underlying computational algorithm and key features. Each of the tools was developed to analyze short-read-based whole-genome sequence data.

**Table 3:** Tools for genotyping VNTRs.

VNTR genotyping tool	Algorithm description	Genotype TRs that exceed the read limit?	Detects TRs not annotated in reference?	Other notes/features
VNTRSeek [66]	Sample TRs are mapped to the reference TRs based on similarity in the repeat consensus patterns, and the TR array profiles. Pairings are confirmed with three types of alignment: (i) longest common subsequence (LCS) comparison of consensus patterns; (ii) profile alignment of TR arrays; and (iii) edit-distance alignment of flanking sequences	No	No	First software developed for genome-wide detection of VNTRs, Each VNTR can be modeled individually, and complex models can be constructed for VNTRs with complex structure, along with VNTR specific confidence scores
adVNTR [65]	Requires training of separate Hidden Markov Models (HMM) models for each combination of target VNTR and sequencing technologies	Yes	No	Provides a uniform training framework, but permits tailoring the models for complex VNTRs on a case-by-case basis
adVNTR-NN [67]	Uses shallow neural networks for fast read recruitment followed by sensitive Hidden Markov Models (HMMs) for genotyping	Yes	No	Novel use of neural networks as a filtering strategy could lead to an order of magnitude reduction in compute time

Several publicly available tools for genotyping VNTRs from whole-genome-sequence data are tabulated, along with notes on the underlying computational algorithm and key features. Each of the tools was developed to analyze short-read-based whole-genome sequence data.

Notably, there are few comprehensive and independent reports of the comparative accuracy of the available TR-calling algorithms, so the selection of algorithms is often application-specific [55, 60, 61]. For profiling common STR variation, computational tools named *HipSTR* and *LobSTR*, can genotype STRs with length less than the sequencing read length (i.e., 100–150 bp Illumina sequencing read), so may be used for high-throughput profiling at annotated STR loci [62, 63]. However, as the majority of rare repeat expansions are greater than 150 bp, these tools are unable to detect most pathogenic TR expansions*. HipSTR* was developed more recently than *LobSTR* and several publications have incorporated *HipSTR* as a singular STR profiling tool, including for example analyses that have genotyped STRs in the Simons Simplex Collection or in the GTEx dataset [57, 64]. In addition, as per some reports, *lobSTR* may be error-prone in sequencing dinucleotide repeats [55]. Relatively fewer NGS-based tools have been developed for VNTR genotyping. Each VNTR genotyping tool varies by underlying computational algorithm, including *VNTRSeek* and *advNTR*, and each with limited application to date [65–67]. The former tool, *VNTRSeek* can also only genotype repeats less than the read length, and there are concerns about the reliability of *adVNTR* calls for longer VNTRs, at higher motif lengths, as per independent long-read validation experiments [68]. There are additional tools designed to enable detection of tandem repeat expansions that exceed the read length (i.e., >100–150 bp Illumina sequencing read), including: *STRetch, gangSTR, TREDPARSE, superSTR, Expansion Hunter*, *exSTRa* and *RepeatSeq*, the latter tool most dated in publication and release [69–75]. Each of these tools can detect expansions at already-annotated, reference TR loci using an alternative underlying computational algorithm, but some may have expansion biases. Other NGS-based TRE detection tools, may detect expansions at unannotated STR loci, such as *STRling* or *ExpansionHunter Denovo* as well as resolve TREs at base pair resolution; however, *STRling* is a relatively new method without robust replication studies to date [76, 77]. Overall, the use of more than one algorithm for genotyping TRs from short-read data and evaluating the convergence of results obtained by using different tools may offer increased reliability and mitigate against false positive calls. Lastly, other than the aforementioned tools specifically developed for genotyping STRs or VNTRs, a suite of other NGS-based tools initially developed to determine read-depth for calling CNVs may alternatively be used for determining repeat units (or copy number) at STR or VNTR loci, such as *CNVnator, CNVpytor, or MosDepth* and therefore have been used in some reports of TR profiling [34, 78–80].

In addition to genotyping TRs from short-read-based WGS data, the evolution and increasing application of long-read sequencing will enable the sequencing of challenging regions of the human genome, such as long TR variation, with increased accuracy compared to short-read sequencing approaches [81]. Long-read technologies are advantageous since they can generate continuous sequences ranging from 10 kilobases to several megabases in length, directly from native DNA [82, 83]. Long-read sequencing has already been used to date to validate short-read-based TR-calling algorithms in some research reports and clinically, for diagnoses of some known TR expansion disorders. Long-read sequencing has also enabled the recent identification of additional novel, TRE disorders, such as familial adult myoclonic epilepsy (FAME) and cerebellar ataxia, neuropathy and vestibular areflexia syndrome (CANVAS) [30, 68, 84]. As the overall reference genome continues to be sequenced with evolving long-read-based approaches (including pangenome graphs and other methods), as exemplified by the “telomere-to-telomere” whole-genome sequencing effort, refinement of annotated, reference structural variant loci, including TR loci, will evolve, with consequent opportunities for refined disease-association [85–87]. For example, long-read sequencing of a modest diversity panel of 15 human genomes identified almost 100,000 structural variants, most previously unknown, including VNTRs shown to be most non-randomly distributed, many mapping to the last 5 Mb of sub-telomeric regions [88].

## Other biological effects of tandem repeat variation

Other than TR effects on phenotype, the effects of common tandem repeat variation at the level of gene expression are increasingly being elucidated across tissue type. In an initial study of lymphoblastoid cell lines, 2060 STRs were found to significantly influence nearby gene expression (i.e., “eSTRs”), contributing 10–15% of *cis*-heritability mediated by all common variants [89]. Subsequent analysis of the Genotype-Tissue Expression (GTEx) cross-tissue repository, identified ~28,000 eSTRs associated with the expression of ~12,500 genes, in 17 tissues, including ~1000 eSTRs in caudate and ~1900 eSTRs in cerebellum, albeit with small sample sizes for limited brain regions [64]. In another cross-tissue analysis, within a subset of ~10,000 genotyped VNTRs, ~160 VNTRs (1.6%) were found to significantly affect gene expression (i.e., “eVNTRs”) across 46 tissue types [67]. An alternative VNTR calling algorithm, genotyped VNTRs genome-wide, reporting 2980 significant eVNTRs (4.2%) across 48 tissues, though the set of brain samples included was limited to 90 frontal cortex and 73 hippocampal samples [68]. The study also identified thousands of VNTRs that significantly influence CpG methylation (“mVNTRs”), with many VNTR loci associated with both expression and methylation. In another epigenetic analysis, of 4849 promoter-associated STRs genotyped in 120 individuals within the HapMap dataset, length polymorphism in >100 TRs was found to effect neighboring gene expression and DNA methylation (“mQTLs”) [90].

Moreover, several studies suggest that tandem repeat variants throughout the genome regulate gene splicing, in which alternative proteins or non-coding products result from alternative splicing of a single gene, that may be differentially expressed, with divergent roles in biological processes or in complex disease [25, 91]. Within SCZ risk loci specifically, as cited above, a VNTR in the 5’ untranslated region of *AS3MT* and in linkage with the GWAS index SNP was found to be associated with the expression of a novel, brain-specific alternative transcript lacking exon 2 and exon 3 compared to the canonical full-length transcript [47]. Likewise, within another SCZ GWAS-positive locus containing the *MIR137HG* gene encoding microRNA-137, novel splice variants that exclude the mature miR-137 sequence were significantly associated with a proximal VNTR element that was also associated with SCZ risk. The novel transcript was found to down-regulate miR-137 expression and to be developmentally regulated [49]. As another example, More systematically throughout the genome, a recent large-scale study, generated a genome-wide catalog of 95,377 tandem repeats regulating gene splicing in *cis* (spl-TRs), including 58,290 significant TR-splicing associations across 49 tissues within GTEx [91]. Two of the identified spl-TRs were within known loci for repeat expansion disorders (spinocerebellar ataxias, *SCA6* and *SCA12*). Overall, the number of significant TR-splicing associations varied by tissue, and brain region, for example, 536 were reported for frontal cortex and 392 for hippocampus, though the mechanism for most spl-TRs and their disease relevance remains unknown. Notably, systematic TR functional genomics reports to date have not been specific to schizophrenia, per se.

Indeed, most post-mortem brain transcriptomic eQTL and mQTL reports focusing on schizophrenia to date, have queried SNPs influencing gene expression, in *cis* and *trans*, but have overlooked consideration of TR elements throughout the genome [92–94]. Future investigation of the role of TRs in SCZ post-mortem brain transcriptomic analyses, including alternative splicing events, is needed to elucidate the potential effects of TRs on gene expression, in mediating SCZ risk.

## Discussion

The current review highlights biological and clinical features of an underexplored but ubiquitous class of genetic variant, tandem repeat variation. Both rare TR expansions and common STR and VNTR variation have been under-assayed in recent, schizophrenia genome-wide association studies, largely due to past technical difficulty in their genotyping, as well as limited access to whole-genome sequenced cohorts. As methods for TR profiling at high throughput emerge, along with the refinement of the (TR) reference genome, and as WGS data for schizophrenia cohorts become increasingly available at reduced sequencing costs, an opportunity for the identification of TR variation associated with schizophrenia risk will become increasingly apparent and feasible. It is conceivable that a fraction of some individuals with schizophrenia or psychosis may harbor rare TR expansions that are “pathogenic”, analogous to carriers of rare “pathogenic” CNVs or single-nucleotide variants, that may then be amenable to identification and stratification from other cases of idiopathic schizophrenia, to enable refined targeted diagnostic and treatment strategies.

In addition, common STR and VNTR elements may contribute to phenotypic risk association, warranting investigation of STR and VNTR elements within schizophrenia GWAS-positive loci, as well as at other novel loci throughout the genome, not yet reported. At the mechanistic level, future schizophrenia transcriptomic and epigenomic analyses may integrate the brain-specific effects of common TR variation with other common genomic variants in influencing gene expression and methylation outcomes, in bulk homogenate tissue as well as single-cell analyses.

The investigation of cell-specificity and somatic mosaicism of schizophrenia-associated TR variation may also be biologically insightful and disease-relevant. Somatic mosaicism of some pathogenic TREs has been demonstrated to influence genotype-phenotype correlation for example, recent work in HD suggests that somatic instability of poly-CAG repeat tracts, which can expand into the hundreds in neurons, explains clinical outcomes better than the length of the inherited allele [95, 96].

Overall, TR variation most likely contributes to numerous complex disorders beyond schizophrenia, including other neuropsychiatric disorders, as has already begun to be addressed in initial autism studies, warranting consideration of the genomic and functional effect of TR variation broadly. That many known pathogenic TR expansions primarily result in disorders of the central nervous system remains an interesting and notable point of pathophysiological specificity. Notably, some structural variant catalogs detailing variant structure and function for general medical and population genetics applications initially omitted inclusion of TR variants, while prioritizing other classes of structural variation, underscoring the difficulty in accurately assaying TRs but also the latent opportunity for elucidating novel factors contributory to complex disease risk [97, 98].
